# Inherited epidermolysis bullosa: update on the clinical and genetic aspects^☆^^☆☆^

**DOI:** 10.1016/j.abd.2020.05.001

**Published:** 2020-07-08

**Authors:** Luiza Monteavaro Mariath, Juliana Tosetto Santin, Lavínia Schuler-Faccini, Ana Elisa Kiszewski

**Affiliations:** a Postgraduate Program in Genetics and Molecular Biology, Universidade Federal do Rio Grande do Sul, Porto Alegre, RS, Brazil; b Postgraduate Program in Child and Adolescent Health, Universidade Federal do Rio Grande do Sul, Porto Alegre, RS, Brazil; c Dermatology Service, Santa Casa de Misericórdia de Porto Alegre/Universidade Federal de Ciências da Saúde de Porto Alegre, Porto Alegre, RS, Brazil; d Department of Genetics, Universidade Federal do Rio Grande do Sul, Porto Alegre, RS, Brazil; e Department of Clinical Medicine, Universidade Federal de Ciências da Saúde de Porto Alegre, Porto Alegre, RS, Brazil; f Pediatric Dermatology Unit, Santa Casa de Misericórdia de Porto Alegre/Universidade Federal de Ciências da Saúde de Porto Alegre, Porto Alegre, RS, Brazil

**Keywords:** Epidermolysis bullosa, Inheritance patterns, Skin diseases,, genetic

## Abstract

Inherited epidermolysis bullosa is a group of genetic diseases characterized by skin fragility and blistering on the skin and mucous membranes in response to minimal trauma. Epidermolysis bullosa is clinically and genetically very heterogeneous, being classified into four main types according to the layer of skin in which blistering occurs: epidermolysis bullosa simplex (intraepidermal), junctional epidermolysis bullosa (within the lamina lucida of the basement membrane), dystrophic epidermolysis bullosa (below the basement membrane), and Kindler epidermolysis bullosa (mixed skin cleavage pattern). Furthermore, epidermolysis bullosa is stratified into several subtypes, which consider the clinical characteristics, the distribution of the blisters, and the severity of cutaneous and extracutaneous signs. Pathogenic variants in at least 16 genes that encode proteins essential for the integrity and adhesion of skin layers have already been associated with different subtypes of epidermolysis bullosa. The marked heterogeneity of the disease, which includes phenotypes with a broad spectrum of severity and many causal genes, hinders its classification and diagnosis. For this reason, dermatologists and geneticists regularly review and update the classification criteria. This review aimed to update the state of the art on inherited epidermolysis bullosa, with a special focus on the associated clinical and genetic aspects, presenting data from the most recent reclassification consensus, published in 2020.

## Introduction

Inherited epidermolysis bullosa (EB) is a group of genetic diseases associated with skin fragility, which leads to the formation of blisters, erosions, and scars on the skin and mucous membranes in response to minimal mechanical trauma.^1^ EB is clinically and genetically very heterogeneous, comprising phenotypes with contrasting levels of severity and involving changes in at least 16 different genes.^2^ Given such heterogeneity, EB is classified into four main types and in several clinical subtypes. The main classification relates to the layer of skin in which the formation of blisters occurs: EB simplex(EBS; intraepidermal layer), junctional EB (JEB; within the lamina lucida of the basement membrane), dystrophic EB (DEB; below the basement membrane), and Kindler's EB (KEB; mixed skin cleavage pattern).^1^, ^2^, ^3^

Other disorders related to skin fragility are classified into separate categories, either because the formation of blisters is a minimal part of the clinical picture or because the cleavage of the skin is very superficial.^2^ Among them, scamous, erosive and kinhyperkeratotic conditions, and connective tissue disorders with skin fragility are noteworthy.^2^ Until the most recent reclassification of EB types, published in 2020, many of these disorders were previously considered subtypes of EB.^2^, ^3^

Epidemiological data about the incidence and prevalence of EB in the world are quite variable in different studies. One of the main research groups on EB, which included a sample of 3,271 patients with EB in the United States, estimated the incidence (between the years 1986‒2002) and the prevalence (year 2002) of the disease in the country to be 19.57 and 11.07 per 1,000,000 individuals, respectively.^4^ In Brazil there are no epidemiological studies on EB; however, DEBRA Brasil (an association to support patients with EB in Brazil) has a record of over 900 patients in the country (unpublished data).

**Figure 1:**
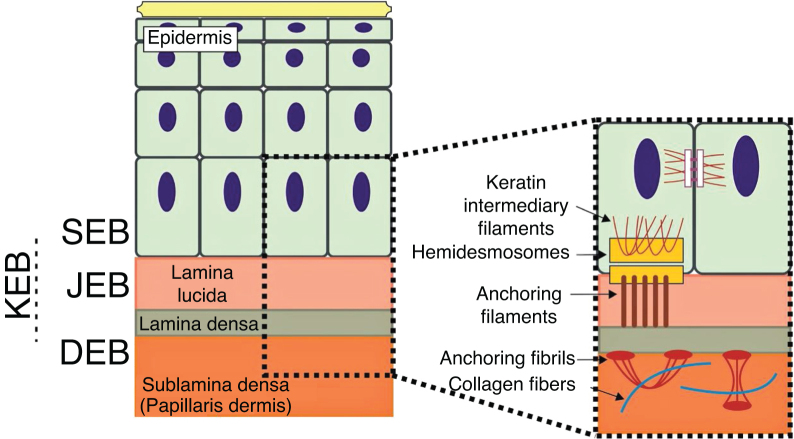
Schematic representation of the skin layers associated with the different types of epidermolysis bullosa (EB). In the epidermis, keratinocytes are depicted. The epidermis is attached to the dermis by the basement membrane, formed by the lamina lucida and lamina densa. On the left side of the figure, each type of EB is presented next to the respective skin layer in which the formation of blisters occurs. Cleavage in EB simplex **(**EBS) occurs within the basal keratinocytes; in junctional EB (JEB), within the lamina lucida; and in dystrophic EB (DEB) occurs in the sublamina densa, in the upper portion of the dermis (papillary dermis). In Kindler's EB (KEB), cleavage can occur in the basal keratinocytes, in the lamina lucida, or below the lamina densa. On the right side of the figure, the main adhesion complexes of the skin layers that are associated with the EB subtypes are shown. Highlighted are hemidesmosomes, anchoring filaments, and anchoring fibrils, which play an essential role in the stable adhesion of the basal keratinocytes of the epidermis to the basement membrane area and for the connection of the lamina densa to the upper portion of the dermis.

The genes associated with EB encode proteins with structural functions in the epidermis, in the basement membrane area or in the upper part of the dermis, being important for the integrity of the skin and for the adhesion between dermis and epidermis.^5^ Thus, genetic changes that alter the dynamics and function of these proteins result in failure of the structures that provide mechanical stability to the epidermis (such as the keratin cytoskeleton and desmosomes) and to the basement membrane area (such as hemidesmosomes, focal adhesions, anchoring filaments, and anchoring fibrils; Fig. 1).^6^ The layer in which the blisters develop in the different types of EB is correlated with the location of the altered protein in the skin structure.

The characteristic phenotype of the different forms of EB is correlated to the gene that is altered. In turn, different genes also lead to very similar EB phenotypes, which adds complexity to the understanding of the associated pathogenic mechanisms.^3^ In addition to the association of certain genes with specific phenotypes, studies have demonstrated the existence of a certain genotype-phenotype correlation with different variants of the same gene. Such results indicate that the nature and position of the pathogenic variant, as well as its consequence in terms of mRNA and protein, are correlated with the clinical manifestations of the disease.^7^, ^8^, ^9^ Phenotypic expressivity in EB is highly variable, not only between the different subtypes, but also within each of them. The EB spectrum ranges from patients with discrete cutaneous signs, often almost imperceptible, to patients with severe cutaneous and extracutaneous lesions, caused by serious involvement of the dermis-epidermis adhesion.^10^

The main clinical sign of EB is the formation of blisters on the skin in areas of mechanical trauma. Depending on the cleavage layer of the skin, the blisters can be more superficial and result in erosions, as in the case of EBS, or they can be deeper and lead to ulcerations, as in the cases of JEB, DEB, and KEB. The blisters may be localized in the extremities or generalized, affecting different parts of the body. Skin lesions can become chronic when mechanical trauma is permanent or recurrent. The oral, esophageal, tracheal, genitourinary, and ocular mucous membranes can also be affected by erosions, ulcers, and scars. In specific EB subtypes, changes in nails and hair can also occur.^3^, ^6^ Progressive healing can result in contractures and/or mutilations of the extremities, microstomy, and esophageal stricture, common features in more severe cases of DEB and KEB. Specific subtypes of EB associated with severe phenotypes may involve other organs and systems, such as osteoporosis, joint contractures, cardiomyopathy, renal amyloidosis, and growth retardation, among others.^3^, ^6^

## Classification criteria for inherited epidermolysis bullosa

The classification of EB is very complex, since it comprises a range of phenotypes with varying levels of severity, associated with changes in a significant number of genes. Thus, specialized EB researchers periodically hold international meetings to develop and update the consensual classification for EB. The most up-to-date classification, published in 2020, indicated a total of 34 distinct subtypes of EB (14 EBS, nine JEB, and 11 DEB), which take into account the skin layer in which the cleavage occurred, the phenotypic characteristics (such as the distribution and severity of the blisters and the presence of specific cutaneous and extracutaneous characteristics), the pattern of genetic inheritance, the expression of the altered protein, and the genetic alteration.^2^ In the next sections, the main types and subtypes of EB will be presented, highlighting the clinical characteristics and genetic bases that constitute the different forms of the disease.^9^

## Epidermolysis bullosa simplex

EBS is characterized by cleavage and blistering at the epidermal level of the skin, being associated with phenotypes with a variable spectrum of severity.^11^, ^12^ EB**S** has a complex genetic background: it is associated with changes in at least seven different genes and, compared to the other main types of EB, it presents the greatest diversity, including at least 14 distinct clinical subtypes.^1^, ^2^, ^3^, ^6^ Most cases of EB**S**are inherited in an autosomal dominant manner, although autosomal recessive inheritance is more common in some regions of the world.^2^

Epidemiological data show that EBS has the highest incidence and prevalence in comparison to the other main types of EB (7.87 and 6.0 per 1,000,000 individuals, respectively), considering a sample from the United States.^4^ The localized form of the disease, which includes milder phenotypes, is the most common subtype of EB**S**(prevalence of 3.9/1,000,000).^4^ Since EBS is often associated with milder clinical phenotypes, it is possible that the disease is underdiagnosed; consequently, the prevalence of this type of EB may be even higher.^12^

### Clinical subtypes of epidermolysis bullosa simplex

EB**S** is the type of EB that comprises the largest number of distinct clinical subgroups. In the latest classification proposed by Has et al., those authors presented a total of 14 subtypes of EB**S** associated with seven different genes.^2^ Table 1 shows the classification of EB**S** subtypes, highlighting the altered gene and protein, as well as the pattern of inheritance.

**Table 1:** Clinical subtypes of epidermolysis bullosa simplex (EB**S**).

Main type (inheritance pattern)	**EBS** subtypes	Gene (protein)
EB**S** (autosomal dominant)	EB**S**, localized	*KRT5*(keratin 5 - K5)
		*KRT14*(keratin 14 - K14)
	EB**S**, intermediate	*KRT5*(keratin 5 - K5)
		*KRT14*(keratin 14 - K14)
	EB**S**, severe	*KRT5*(keratin 5 - K5)
		*KRT14*(keratin 14 - K14)
	EB**S** with mottled pigmentation	*KRT5* (keratin 5 - K5)
	EB**S**, migratory circinate	*KRT5*(keratin 5 - K5)
	EB**S**, intermediate	*PLEC*(plectin)
	EB**S**, intermediate with cardiomyopathy^d^	*KLHL24*(Kelch-like 24)
EB**S**(autosomal recessive)	EB**S**, intermediate or severe	*KRT5*(keratin 5 - K5)
		*KRT14*(keratin 14 - K14)
	EB**S**, intermediate	*PLEC*(plectin)
	EB**S**, localized or intermediate with BP230 deficiency	*STD* (bullous pemphigoid antigen 1 - BP230^a^)
	EB**S**, localized or intermediate with exophyllin-5 deficiency	*EXPH5*(exophilin-5^b^)
	EB**S,**intermediate with muscular dystrophy^d^	*PLEC*(plectin)
	EB**S**, severe with pyloric atresia^d^	*PLEC*(plectin)
	EBS, localized with nephropathy^d^	*CD151* (CD151 antigen^c^)

Table modified from the classification by Has et al. (2).

In bold, the three most prevalent EB**S** subtypes.

a BP230, BPAG1e and dystonin are synonymous.

b Exophilin-5 and Slac2-b are synonymous.

c CD151 antigen and tetraspanin 24 are synonymous.

d Syndromic subtypes of EB**S.**

**Figure 2:**
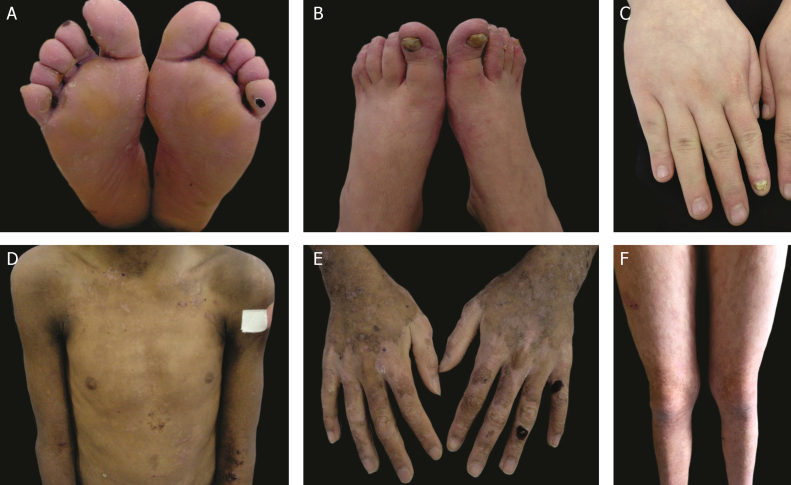
Clinical aspects of epidermolysis bullosa simplex (EBS). (A‒C) Plantar keratoderma, dystrophic and thick nails on the hands and feet of patients with localized EBS. (D‒F) Blisters and erosions distributed in a generalized way in patients with intermediate EBS**.** The presence of epidermolysis bullosa nevi is common in all subtypes of EBS.

The localized, intermediate, and severe forms are the most frequent in EB**S**, with a prevalence of 4.69 per 1,000,000 together, while all other forms of EB**S** together reach a prevalence of 1.31 per 1,000,000.^4^ The three main subtypes are caused by pathogenic variants in the *KRT5* or *KRT14* genes, which encode keratins 5 and 14, respectively, and account for 60%‒70% of the cases of EBS.^1^ Localized EBS, formerly known as Weber-Cockayne EBS, is characterized by blisters limited to the regions of the palms and soles of the feet. The lesions can also appear in other regions of recurrent trauma, and they tend to get worse in warmer months. The blisters are usually not present at birth, but they appear early in childhood, especially when the child begins to crawl and walk. Focal palmoplantar keratoderma (thickening of the skin of the hands and feet) may be observed in some cases during adulthood. Among the possible complications, infections secondary to blistering lesions on the feet are the most common (Fig. 2).^3^, ^11^, ^12^

Severe EBS, formerly known as severe generalized or Dowling-Meara EBS, has a unique clinical phenotype, with large generalized blisters, which can occur in a group (herpetiform blisters) and can be hemorrhagic.^12^ During childhood, blisters develop throughout the body, most often reaching the hands and feet, the region around the mouth, and the regions of the trunk and neck. In this subtype, the involvement of the oral mucosa, the occurrence of progressive palmoplantar keratosis, and nail dystrophy are common.^11^ Lesions commonly heal without scarring, but inflammation can occur, especially in hemorrhagic blisters, followed by milia and hypo- and hyper-pigmentation of the skin. As in the other EB**S** subtypes, the blisters tend to improve with age; however, palmoplantar keratoderma is more severe in most cases (Fig. 2).^3^, ^11^, ^12^

Intermediate EBS, formerly known as intermediate generalized EB**S**or KoebnerEBS, often manifests at birth, with the generalized distribution of blisters, but without the formation of herpetiform clusters. The clinical presentation is milder in comparison with the severe form, and does not present extracutaneous involvement. Although the distribution is widespread, the blisters predominantly affect the hands and feet. Focal palmoplantar keratoderma can be observed, and lesions tend to get worse with heat.^3^, ^11^, ^12^

The three main subtypes of EBS, detailed above, are caused by changes in the genes encoding keratins 5 and 14 of the keratinocyte cytoskeleton, as will be further detailed in the next section. In addition to them, other forms of EB**S** are also noteworthy, because they represent a considerable fraction of all cases. Pathogenic variants in the *PLEC*gene, which encodes the plectin protein, can result in different subtypes with different inheritance patterns, which together account for up to 8% of cases of EBS.^1^, ^13^ The autosomal dominant form results in intermediate EBS, while the recessive form can result in intermediate EBS, intermediate EB**S**with muscular dystrophy, and severe EB**S**with pyloric atresia.^2^ IntermediateEB**S**with muscular dystrophy is characterized by the occurrence of blisters since birth and progressive muscle weakness with late onset.^14^, ^15^ The main characteristics of severe EB**S** with pyloric atresia are the occurrence of blisters in the neonatal period, the presence of pyloric atresia and, frequently, premature death.^15^, ^16^ In contrast, the subtype with autosomal dominant inheritance, formerly known as Ogna EBS, which is now just intermediate EBS, is characterized by a mild clinical picture, which includes mild skin fragility and the absence of involvement of other organs.^15^, ^17^, ^18^

In 2016, two independent studies almost simultaneously identified pathogenic variants in the *KLHL24*gene, encoding the Kelch-like protein 24, in a total of 15 patients with the EB-compatible phenotype who were not related to each other.^19^, ^20^ Subsequently, other researchers began to investigate possible variants in this gene in patients who remained until that moment without the identified genetic mutation. Several cases with typical signs of EB and mutations in *KLHL24*have been described since then.^21^, ^22^, ^23^, ^24^, ^25^ It is estimated that 5% of cases of EB**S**are associated with mutations in this gene.^1^ The typical clinical signs of this subtype of EB**S** are still being characterized, as more studies and more patients are being described. Initial reports indicated that the phenotype would almost exclusively involve skin fragility, with lesions since birth, especially in the legs, and the formation of blisters on the trunk and upper limbs.^21^ From additional reports, a more syndromic phenotype appears to be related, with extracutaneous involvement that would include a strong association with cardiomyopathy.^23^, ^24^ Studies have demonstrated that 85% of patients with pathogenic variants in *KLHL24*have some cardiac involvement, evidenced by a high level of biomarkers or dilated cardiomyopathy (40%).^25^, ^26^ Neurological problems, such as developmental delay, intellectual disability, memory problems, and myopathy were also described in some cases.^26^ Future studies will be indispensable for a complete clinical characterization of this new subtype of EBS, designated in the most recent classification of EB as intermediateEB**S** with cardiomyopathy.^2^

The other subtypes of EB**S** described in Table 1 are considered rare, none of which represents more than 1% of the total cases of EBS.^1^ Some of them have specific clinical signs, such as the presence of mottled pigmentation and circinate plaques of a migratory character in the subtypes that bear these names. Other subtypes are characterized by clinical signs with different levels of severity, which overlap with other forms of the disease.^3^ For these cases, the clinical examination is not sufficient for the diagnosis, and additional techniques are needed to identify the altered protein or the pathogenic genetic variant.^27^

### Genetics of epidermolysis bullosa simplex

**Figure 3:**
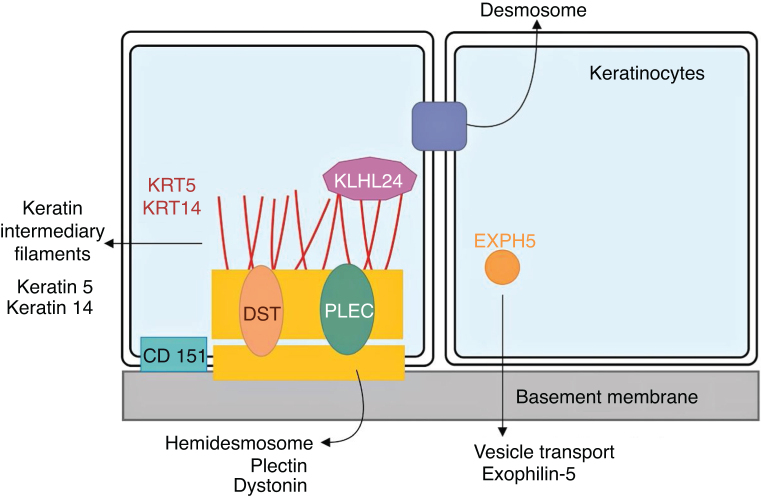
Schematic representation of the complexes and proteins associated with the different subtypes of epidermolysis bullosa simplex (EBS). Changes in structural components of hemidesmosomes, intermediate keratin filaments, and cell vesicle transport support the spectrum of EBS phenotypes. Only proteins associated with EBS are shown in the figure.

Although clinical expressivity varies between different forms of EBS, almost all are caused by changes in genes that encode components of keratinocytes that are important for the organization of the cytoskeleton and for cell-cell junctions.^12^, ^28^ Thus, most of the proteins associated with EBS, detailed below, have the main function of providing structural support to the keratinocytes of the epidermis (Fig. 3).^12^

### Keratins 5 and 14

**Figure 4:**
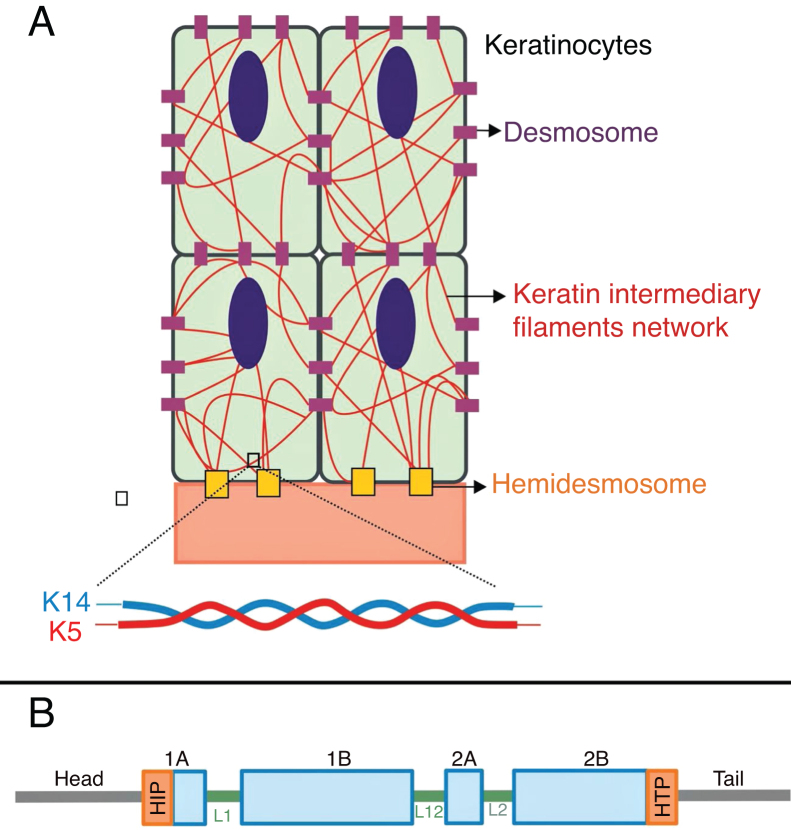
(A) Schematic representation of the keratin intermediate filament network. The keratin filaments connect to the hemidesmosomes to ensure attachment to the underlying basement membrane and to the desmosomes to ensure cell-cell contact in the keratinocytes. A representation of the molecular configuration of the K5/K14 heterodimer, the smallest subunit that forms the intermediate filaments, is shown at the bottom of figure A. (B) Organization of keratin domains. Keratins have a central α-helix rod domain containing four segments (1A ‒ B, 2A ‒ B), which are interrupted by three binding domains (L1, L12, and L2). The rod domain has highly conserved motifs (HIP and HTP) at its ends, often associated with the most severe cases of severe EBS. Variants in the binding domains are generally associated with localized EBS, while in the intermediate form of the disease the variants involved tend to be distributed across segments of the rod domain.

Keratins are cytoskeletal proteins that provide mechanical stability to cells and epithelial tissues. The keratin filaments are inserted between the desmosomes and hemidesmosomes, forming a network that allows not only the cell-cell connection of the keratinocytes, but also between them and the underlying layer of the dermoepidermal junction.^11^, ^12^ Keratins 14 (K14) and 5 (K5) are type I and II intermediate filament proteins, respectively, and are expressed in the basal keratinocytes in the epidermis. In the physiological state, K14 and K5 form heterodimers of type I/type II intermediate filaments; thousands of them group together to form the cytoskeleton of intermediate keratin filaments (Fig. 4A).^12^

Pathogenic variants in the *KRT14*and *KRT5* genes, which code K14 and K5, respectively, impair the assembly of the intermediate filament network in the keratinocyte cytoplasm, resulting in the EBS phenotype.^8^ Amino acid substitutions (missense variants) are the main type of genetic alteration in these genes, and the pattern of inheritance is autosomal dominant, with some exceptions. The clinical severity of EBS is correlated, in most cases, to the location of the genetic changes. Thus, variants in the highly conserved ends of the α-helix rod domains, essential for the assembly of the keratin filaments, are often associated with the most severe cases of the disease (severe EBS, Fig. 4B).^8^, ^29^ However, this correlation is not true for all cases, as some residues may be more or less important for the protein, and the nature of the substituted amino acids, such as their structure and polarity, also influences the phenotype.^8^, ^30^, ^31^, ^32^ The milder form, localized EBS, is commonly associated with variants in the linker region, while the intermediate form (intermediate EBS) comprises variants that tend to be distributed along the K5 and K14 sequences.^8^, ^30^, ^33^

### Plectin

Plectin, coded by the *PLEC*gene, is a binding protein that plays an essential role in stabilizing and organizing the keratin filament networks in the cells. Although it is able to interact with actin filaments and microtubules, the main interaction of plectin is with the filaments of keratin intermediates. It works by anchoring the intermediate filaments to strategic sites, such as focal adhesions, desmosomes, hemidesmosomes, and other adhesion structures, thus stabilizing the network and cells.^34^, ^35^ There are several isoforms of *PLEC*, which are preferentially expressed in different specific tissues.^15^

Pathogenic variants in the *PLEC*gene can lead to different subtypes of EBS: a) intermediate EBS with autosomal dominant inheritance pattern; b) intermediate EBS with autosomal dominant inheritance pattern; c) intermediate EBS with muscular dystrophy; d) severe EBS with pyloric atresia – the last two also have a recessive inheritance pattern.^2^ To date, no clear genotype-phenotype correlation has been established regarding the position of the variants in *PLEC*. Most cases of EBS with muscular dystrophy and EBS with pyloric atresia involve substitutions that lead to a premature stop codon (nonsense variants) or insertions/deletions that alter the reading phase (frameshift variants), in both cases resulting in reduction or complete absence of plectin expression.^34^ It is suggested that the effect of the variants on the different isoforms could explain the phenotypic difference between these two subtypes (EBS with muscular dystrophy and EBS with pyloric atresia).^34^ To date, the only dominant pathogenic variant known to be associated with intermediate EBS (formerly Ogna EBS) is c.5998C > T (p. Arg.2000Trp), which leads to the disturbance of plectin by a negative dominant effect.^18^, ^34^, ^36^ Simply stated, variants with a negative dominant effect are those that lead to the formation of an altered protein that negatively affects the normal wild-type protein that is coexpressed within the cell, reducing its cellular function.

### Other associated proteins and the Kelch-like protein

Most of the seven genes associated with EB**S**have structural functions for organizing the layers of the skin. The *DST*gene, which encodes epidermal dystonin, also known as bullous pemphigoid antigen (BP230 or BPAG1e), plays a role in the regulation of keratinocytes adhesion and in the control of the integrin subtypes expressed.^37^ The exophilin-5 protein, encoded by *EXPH5*, is not an integral component of the intermediate filaments, desmosomes, or hemidesmosomes. Although its role is not fully known, it is suggested that it contributes to the regulation of cell membrane traffic, including controlling the formation and movement of vesicles along the actin and tubulin networks.^37^

Since the discovery of the association of the *KLHL24*gene with the EBS phenotype, researchers have sought to understand the molecular mechanism involved in its pathogenicity.^20^, ^26^, ^38^ Unlike most EBS-associated proteins, the Kelch-like protein 24 is not a structural protein. All pathogenic variants identified in patients involve the start codon of the translation.^26^ Preliminary findings suggest that this protein would act by a new mechanism in EB, through the deregulation of its autoubiquitination, which would lead to changes in the levels of the intermediate filaments, especially keratin 14, in keratinocytes.^20^, ^25^, ^38^

## Junctional epidermolysis bullosa

JEB is characterized by cleavage and blistering within the lamina lucida of the skin. The dermoepidermal separation in this type of EB results from genetic alterations that affect the functions of essential components of the basement membrane zone.^39^, ^40^, ^41^, ^42^ JEB is associated with a broad phenotypic spectrum, ranging from one extreme characterized by early lethality to another extreme with clinical signs so subtle that they are almost indistinguishable from the milder subtypes of EB.^3^ Mutations in seven different genes lead to different subtypes of JEB, all associated with the pattern of autosomal recessive inheritance.^2^, ^3^

Compared to the other main types of EB, JEB is the rarest form of the disease, with an estimated incidence of 2.68 per 1,000,000 live births and a prevalence of 0.5 per 1,000,000.^4^ Since the most severe forms are associated with early lethality, incidence and prevalence estimates are likely to be underestimated.^4^

### Clinical subtypes of junctional epidermolysis bullosa

JEB is classified into nine different subtypes, according to the clinical signs presented and the severity of the phenotype. The most frequent subtypes of JEB are caused by pathogenic variants in the genes encoding the laminin 332 protein and type XVII collagen. The type of genetic alteration and its consequence for protein expression are directly linked to phenotypic severity, as will be discussed in the next section. Table 2 presents the JEB subtypes with emphasis on genes and associated proteins.^2^

**Table 2:** Clinical subtypes of junctional epidermolysis bullosa (JEB).

JEB subtypes^a^	Gene (protein)
JEB, severe	*LAMA3*, *LAMB3*, *LAMC2*(laminin 332)
JEB, intermediate	*LAMA3*, *LAMB3*, *LAMC2*(laminin 332)
JEB, intermediate	*COL17A1*(collagen XVII)
JEB with pyloric atresia^b^	*ITGB4*, *ITGA6*(α6β4 integrin)
JEB, localized	*COL17A1*(collagen XVII)
	*ITGB4*(α6β4 integrin)
	*LAMA3*, *LAMB3*, *LAMC2*(laminin 332)
	*ITGA3*(integrin α3 subunit)
JEB, inversa	*LAMA3*, *LAMB3*, *LAMC2* (laminin 332)
JEB, late onset	*COL17A1*(collagen XVII)
JEB, LOC syndrome	*LAMA3A*(α3A laminin)
JEB with interstitial lung disease and nephrotic syndrome^b^	*ITGA3* (integrin α3 subunit)

Modified table from Has et al., 2020 (2).

In bold, the most frequent JEB groups.

LOC, laryngo-onycho-cutaneous.

a All JEB subtypes have autosomal recessive inheritance.

b JEB syndromic subtypes.

**Figure 5:**
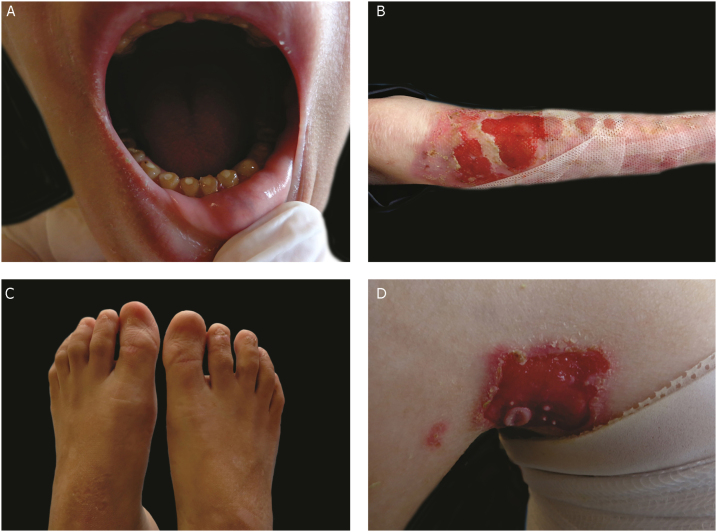
Clinical aspects of junctional epidermolysis bullosa (JEB). Dental enamel anomalies, chronic wounds, and absence of nails in a patient with severe JEB.

The most severe form of this group is severe JEB, previously known as generalized severe JEB or Herlitz JEB, which is characterized by a clinical presentation of extensive skin and mucous membranes fragility.^2^, ^3^ Mucocutaneous blisters and erosions are present since birth and are observed throughout the body’s surface, resulting in loss of proteins, fluids, and iron, which increases susceptibility to infections (Fig. 5).^9^ Exuberant granulation tissue is formed, especially around the nose and mouth, on the buttocks, and on the nail folds.^43^ Patients suffer from extreme pain, and the long-term consequences include developmental delay, difficulty in healing wounds, anemia, respiratory complications, and infections that eventually lead to death in the first years of life.^9^, ^41^, ^44^ This JEB subtype is associated with the complete absence or profound reduction of laminin 332, as will be detailed in the subsequent section.^3^, ^9^

The sub-type intermediate JEB, formerly known as generalized intermediate JEB or generalized non-Herlitz JEB, comprises a clinically heterogeneous group, with generalized distribution of blisters, which heal and leave subtle atrophy and hypopigmentation at the injury sites (Fig. 5).^2^ The severity of the disease is milder when compared with the most severe subtype, although the membranes can also be affected.^9^ Other manifestations include alopecia, enamel defects, and dystrophy or absence of nails, at different levels of severity. Granulation tissue, formation of chronic wounds, and extracutaneous involvement in the cornea, larynx, and urinary tract may also be associated.^3^, ^9^, ^41^, ^42^ In this JEB subtype, the expression of laminin 332 or type XVII collagen is reduced, but not entirely absent.^9^, ^45^

The localized form of JEB was considered a rare condition. The improvement of molecular analysis techniques allowed the identification of cases with atypical clinical presentation in relation to the most well-known EB subtypes and, since then, a series of new cases of localized JEB have been described.^42^, ^45^, ^46^, ^47^ In this subtype, the blisters are locally distributed, mainly in the limbs. Localized JEB, as well as the intermediate subtype, is associated with dystrophy or absence of nails, hypoplasia of tooth enamel, and the tendency to develop cavities (Fig. 5).^3^, ^48^ In contrast to the other subtypes, patients with this form of JEB usually do not have extensive atrophic scars, alopecia, and other extracutaneous findings such as anemia, developmental delay, ocular alterations, or respiratory and genitourinary changes.^3^, ^48^ Phenotypic variability is striking in JEB; in contrast to the severe form of the disease, the localized form may present a phenotype so mild that the only clinical sign is nail dystrophy.^45^

Among the rarest subtypes of JEB, the inversa form stands out, in which the cutaneous manifestation occurs predominantly in intertriginous areas (areas of the skin that touch or rub) and usually more extensive than to localized JEB.^48^ The subtype JEB with pyloric atresia is associated with alterations in the genes encoding the α6β4 integrin and is associated with severe phenotypes, which include, in addition to pyloric atresia, generalized distribution of blisters, congenital absence of skin, and early lethality.^3^, ^49^ However, JEB with pyloric atresia is clinically heterogeneous and is also associated with less severe cases.^50^

### Genetics of junctional epidermolysis bullosa

The dermoepidermal junction area comprises a specialized basement membrane that connects the epidermis to the dermis and thus provides the skin with integrity and resistance against external mechanical forces.^9^, ^51^ The membrane is formed mainly by networks of laminin 332 and collagen IV, which overlap and connect by the action of other molecules.^9^, ^52^, ^53^ Several proteins of the cell surface of the keratinocytes and the extracellular matrix are inserted in this network and interact in a specific way with each other, ensuring the adhesion of the skin layers.^9^, ^52^, ^54^

Pathogenic variants in the genes that encode the components of the dermoepidermal junction result in the absence or disturbance of their functions and the consequent decrease in adhesion of the skin layers, which leads to the characteristic JEB phenotypes.^9^, ^52^

### Laminin 332

**Figure 6:**
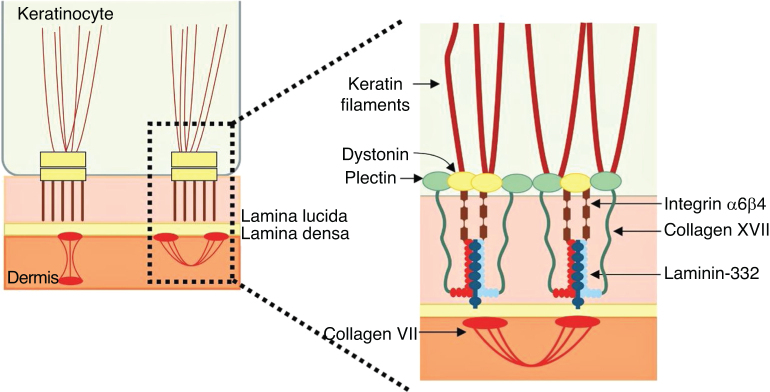
Schematic representation of the main structures involved in the adhesion of the skin layers. The main proteins associated with junctional epidermolysis bullosa ( to the right of the figure) interact in order to allow the connection of the keratin intermediate filaments to the anchoring fibrils (formed by type VII collagen) in the dermis.

Laminin 332, formerly known as laminin 5, is a heterotrimeric protein consisting of α3, β3, and γ2 chains, encoded by the *LAMA3*, *LAMB3*, and *LAMC2*genes, respectively. It is one of the essential proteins of the dermoepidermal junction, being considered a molecular bridge between the basal keratinocytes of the epidermis and the underlying dermis.^9^, ^55^ To connect to the surface of basal keratinocytes, laminin 332 interacts with α6β4 integrin in hemidesmosomes and α3β1 integrin in focal adhesions. On the dermal side, the connection to the basement membrane is due to its interaction with type VII collagen in the anchoring fibrils, which ensures the adhesion of the basement membrane to the extracellular matrix of the dermis (Fig. 6). It is through these protein-protein interactions that laminin 332 acts as a support for dermoepidermal adhesion.^9^, ^41^, ^52^

Pathogenic variants in the *LAMA3*, *LAMB3*, or *LAMC2*genes lead to the absence or reduction of laminin 332, which results in the loss of its function as a bridge between hemidesmosomes and anchoring fibrils. Consequently, the epidermis and dermis separate at the level of the lamina lucida, resulting in typical JEB phenotypes.^52^ The phenotypic severity and, therefore, the clinical subtype of JEB are related to the expression of laminin 332. The most severe form, severe JEB, is usually caused by loss of function variants (null variants), such as the substitutions that generate a premature stop codon (nonsense variants) and those that alter the reading phase (frameshift variants), thus resulting in the complete absence of laminin 332.^9^, ^41^

In approximately 80% of severe cases, the associated gene is *LAMB3*, which encodes the protein's β3 chain.^41^ In the milder forms of JEB (intermediate and localized), at least one of the associated pathogenic variants allows the expression, even if partial, of the functional protein, such as amino acid substitutions (missense) or variants in splice sites that do not alter the reading phase (in-frame).^9^, ^39^, ^40^

### Collagen XVII

Type XVII collagen, encoded by the *COL17A1*gene, is a type II-oriented transmembrane protein, in which the N-terminal end is cytoplasmic and the C-terminal crosses the lamina lucida towards the outer side of the cell.^46^ This protein is a three-chain α1 trimer, whose main function is to connect the intermediate keratin filament network to the basement membrane through interactions with the proteins plectin, BP230, and α6β4 integrin (Fig. 6).^45^, ^46^, ^56^

Pathogenic variants in the *COL17A1*gene result in a variable spectrum of phenotypes. Although several studies have searched for possible genotype-phenotype correlations, the only clear correlation is that collagen XVII expression levels directly influence clinical expression. Thus, patients with genetic variants that lead to the complete absence of protein in the skin, such as nonsense and frameshift, present a severe phenotype. In turn, patients with milder symptoms of the disease usually carry missense variants or variants in the in-frame splice sites, which reduce, but do not eliminate collagen XVII expression, or even alter the protein without affecting its expression.^45^, ^46^, ^57^

### A6β4 and α3β1 integrins

A6β4 integrin is a heterodimeric transmembrane polypeptide, located in the center of hemidesmosomes (Fig. 6).^58^ Its main function is to connect the hemidesmosomal plaque of the basal keratinocytes to the underlying basement membrane, thus providing mechanical resistance to the skin and mucous membranes.^58^, ^59^ The phenotypes resulting from pathogenic variants in the *ITGA6*and *ITGB4*genes, which encode the α6β4 integrin, are also quite heterogeneous. Lethal forms of the disease usually result from null mutations (such as nonsense substitutions, and frameshift insertions and deletions) in both alleles, while the milder forms usually involve the presence of amino acid substitutions (missense variants) in at least one of the alleles.^50^

The gene encoding the α3 subunit of the α3β1 integrin has recently been associated with a rare form of JEB (JEB with respiratory and renal involvement).^60^ Although the importance of α3β1 integrin to the epithelium is known, including epidermal keratinocytes, alveolar epithelial cells, and podocytes, the molecular mechanisms involved in this JEB subtype are not yet established.^61^

## Dystrophic epidermolysis bullosa

DEB is characterized by cleavage of the skin in the region of the sublamina densa, in the upper portion of the dermis.^3^ DEB can be inherited with an autosomal dominant as well as an autosomal recessive pattern. It is associated with a wide phenotypic and severity spectrum, ranging from the isolated occurrence of mild nail dystrophy to the generalized formation of blisters with mutilating scarring of the hands and feet, severe extracutaneous involvement, and premature death.^3^, ^7^, ^62^ Although most pathogenic variants of DEB are typical of the dominant or recessive inheritance pattern, some genetic changes have already been described in both forms, adding complexity to the understanding of the molecular basis of this type of EB.^3^, ^63^ The *COL7A1*gene, which encodes the main constituent of anchoring fibrils, type VII collagen, is the only known gene associated with DEB.

DEB is classified into two main groups according to the associated inheritance pattern: dominant DEB (DDEB) and recessive DEB (RDEB). It can be subdivided into at least 11 clinical subtypes, which also take into account the phenotypic characteristics involved.^2^ The incidence and prevalence of DEB were estimated at 6.65 and 3.3 per 1,000,000 live births, respectively, and the prevalence of the dominant and recessive forms is quite similar (1.5 and 1.4 per 1,000,000, respectively); the prevalence of DEB cases without a defined inheritance pattern is 0.4 per 1,000,000.^4^

### Clinical subtypes of dystrophic epidermolysis bullosa

**Figure 7:**
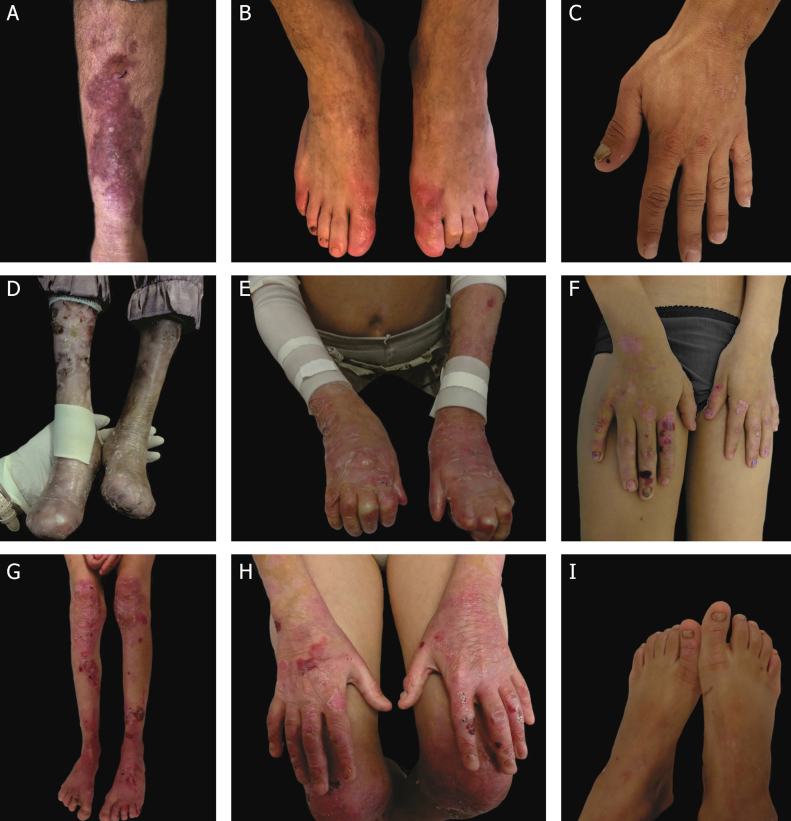
Clinical aspects of dystrophic epidermolysis bullosa (DEB). (A) Patient with dominant DEB, pre-tibial. (B‒C) Dystrophy of the toenails and hands of patients with dominant DEB, localized. (D‒E) Pseudosyndactyly (fusion of the fingers) and generalized distribution of blisters and extensive scarring in a patient with recessive DEB, severe. (G‒H) Blisters and generalized scarring, especially on knees, legs, feet, and hands in a patient with recessive DEB, intermediate; hyperkeratosis of the hands and feet leads to flexion contractures. (F, I) Blisters and lesions restricted to hands and feet and nail dystrophy in patients with recessive DEB, localized.

The classification of DEB in its various subtypes takes into account the characteristic cutaneous and extracutaneous signs, the distribution of the blisters (localized or generalized), and the severity of the phenotype.^2^, ^3^ Although some subtypes are characterized by specific clinical signs of different severity, there is an overlap of certain phenotypes among some groups (Fig. 7A‒I), which makes the diagnosis difficult and requires additional molecular tests.^3^, ^27^ As DEB is inherited in both a dominant and a recessive manner, the classification of the type of DEB is especially important for the purposes of genetic counseling.^6^ Table 3 presents the DEB subtypes.

**Table 3:** Clinical subtypes of dystrophic epidermolysis bullosa (DEB).

Main type	DEB subtypes	Gene (protein)
Dominant DEB (DDEB)	**DDEB, intermedi**ate	*COL7A1*(type VII collagen)
	**DDEB, localized**^a^	*COL7A1*(type VII collagen)
	DDEB, pruriginosa	*COL7A1* (type VII collagen)
	DDEB, self-improving^b^	*COL7A1*(type VII collagen)
Recessive DEB (RDEB)	**RDEB, severe**	*COL7A1*(type VII collagen)
	**RDEB, intermediate**	*COL7A1*(type VII collagen)
	RDEB, inversa	*COL7A1*(type VII collagen)
	RDEB, localized^c^	*COL7A1*(type VII collagen)
	RDEB, pruriginosa	*COL7A1*(type VII collagen)
	RDEB, self-improving^b^	*COL7A1*(type VII collagen)
Dominant and recessive DEB (compound heterozygosity)	DEB, severe	*COL7A1*(type VII collagen)

Modified table from Has et al., 2020 (2).

In bold, the most prevalent DEB subtypes.

a The DDEB localized subtype includes the subtypes previously known as DDEB pre-tibial, DDEB nails-only, and DDEB acral.

b This subtype was previously known as transient bullous dermolysis of the newborn.

c The RDEB localized subtype encompasses the subtype formerly known as RDEB pre-tibial.

In the DEB subtypes, the severity of the phenotypes is related, at a certain level, to the consequence of *COL7A1*mutations for the formation of anchoring fibrils, which may present morphological changes, have their expression reduced, or even be completely absent, as detailed in the next section.^64^ Although the severe forms of DEB are described as the most frequent, it is possible that there is underdiagnosis of the localized forms, given the involvement of more subtle clinical signs. From the development of new generation sequencing technologies, new studies, including large sample numbers, have enabled the establishment of genotype-phenotype correlations for the disease.^34^, ^35^, ^36^, ^37^, ^38^, ^39^, ^40^, ^41^, ^42^, ^43^, ^44^, ^45^, ^46^, ^47^, ^48^, ^49^, ^50^, ^51^, ^52^, ^53^, ^54^, ^55^, ^56^, ^57^, ^58^, ^59^, ^60^, ^61^, ^62^, ^63^, ^64^, ^65^, ^66^, ^67^ The main subtypes of DEB are detailed below.

Severe RDEB, formerly known as severe generalized RDEB or Hallopeau-Siemens RDEB, constitutes the most severe form of DEB, usually associated with the complete absence of collagen VII expression.^68^ The classic clinical signs are the formation of generalized blisters throughout the body from birth and mutilating scars that lead to pseudosyndactyly (fusion of the fingers) in the hands and feet and that result in joint contractures. Furthermore, chronic ulcers that are difficult to heal, milia, and nail dystrophy are other typical signs (Fig. 7 D‒E). The blisters can be observed in the oral mucosa, in the corneal region, and in the gastrointestinal epithelium; esophageal stenosis is described in many cases. Anemia, developmental delay, and squamous cell carcinoma associated with early death remain components of the pattern of this subtype of RDEB.^3^, ^62^, ^64^

Intermediate RDEB, formerly known as intermediate generalized RDEB or non-Hallopeau-Siemens RDEB, is also characterized the generalized distribution blisters; however, it is less severe (Fig. 7 G‒H).^69^ Very frequent clinical signs of the severe form, such as esophageal constriction, corneal lesions, and pseudosyndactyly of the hands and feet still occur, but are less common in the intermediate form of the disease. Developmental delay and anemia are even more rare conditions in this subtype of RDEB.^3^, ^69^

Among the dominant subtypes, the intermediate form, intermediate DDEB (formerly generalized DDEB), is characterized by the formation of blisters with generalized distribution at birth, albopapuloid lesions, milia, atrophic scarring, and nail dystrophy. In contrast to severe RDEB, developmental delay, severe anemia, and risk of squamous cell carcinoma are not characteristic of the DDEB subtypes.^3^, ^69^

Amid the complexity that is the classification of DEB in its various subtypes, the localized forms of both patterns of inheritance make it even more difficult: localized DDEB and localized RDEB are clinically indistinguishable.^3^ That is, for typical phenotypes of localized DEB, the genetic result is essential for the determination of the associated inheritance pattern and, ultimately, for a correct genetic counseling. These are subtypes with very attenuated EB phenotypes, whose main clinical sign is the localized distribution of blisters in the hand and foot regions and nail dystrophy (Fig. 7 B, C, F, and I). Milia and atrophic scars can also occur. There is no extracutaneous involvement or pseudosyndactyly in these forms of the disease.^3^, ^69^

The DEB subdivision still encompasses a number of other, rarer subtypes, which usually involve specific clinical signs. Inverse RDEB is characterized by a peculiar succession of clinical findings, which begins with the formation of generalized blisters in the neonatal period that heal in early childhood, leaving atrophic scars. Skin signs tend to improve over the years, while serious lesions develop on the oral, esophageal, anal, and genital mucosa.^27^, ^70^ DEB pruriginosa is a generalized subtype that includes the complaint of severe pruritus.^69^, ^71^ DEB self-improving, formerly known as transient bullous dermolysis of the newborn, is a subtype of DEB that appears at birth, or soon after, with the formation of generalized blisters. In contrast to the other subtypes, the clinical signs of this form of the disease disappear within 6 to 24 months of life.^69^

## Genetics of dystrophic epidermolysis bullosa

The dystrophic form of EB is characterized by the separation of the tissue just below the lamina densa, at the level of the anchoring fibrils. As will be detailed, the main constituent of anchoring fibrils is collagen VII, encoded from the *COL7A1*gene. Changes in this gene result in morphological changes or reduction/absence of anchoring fibrils, leading to different DEB phenotypes.^72^ The level of expression of *COL7A1*in patients with DEB is inversely correlated with phenotypic severity.^7^, ^73^

### Type VII collagen

Type VII collagen is the main, if not the only, component of anchoring fibrils. These are large U-shaped structures that extend from the bottom of the dermal-epidermal basement membrane to the upper layer of the dermis (papillary dermis).^52^ Thus, the anchoring fibrils provide integrity for the association of the epidermis with the underlying dermis.^62^

*COL7A1*is a gene that has a complex arrangement, consisting of 118 exons and a size of approximately 32 kb. This gene encodes collagen polypeptides pro-α1 (VII), which will be organized into anchoring fibrils.^62^, ^74^ The structure of these polypeptides includes a collagenous domain in its central portion, formed by Gly-X-Y repeats that fold to achieve the characteristic triple helix conformation of collagen molecules.^62^ The triple helix domain of collagen VII contains a total of 19 disruptions or imperfections, including a non-collagenous central segment of 39 amino acids.^74^ Such interruptions in the Gly-X-Y sequence provide flexibility for collagen VII molecules.^52^ The central collagenous domain is flanked by non-helical globular domains, the NC1 domain at the amino-terminal end and the NC2 domain at the carboxy-terminal end.^52^, ^62^, ^74^

**Figure 8:**
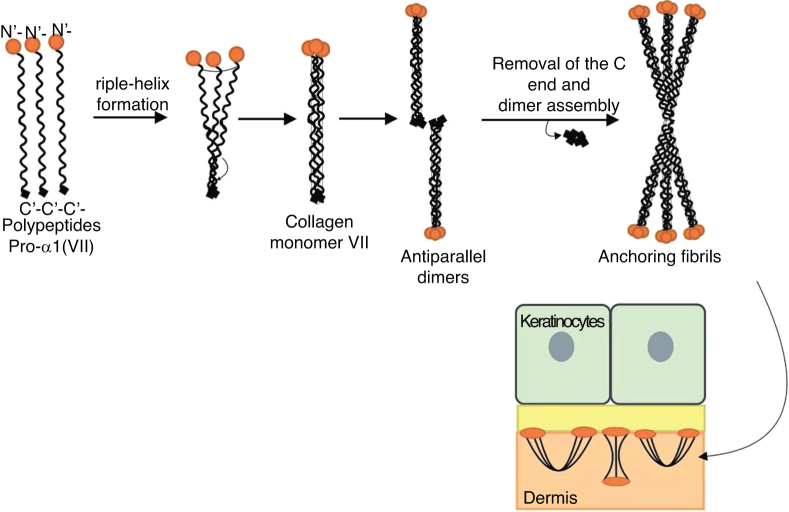
Schematic representation of the assembly of the anchoring fibrils from pro-a1 (VII) collagen polypeptides. Three formed polypeptides are associated through their C-terminal ends, while their collagenous domains fold into a triple helix conformation. Thereafter, type VII collagen monomers in triple helix form antiparallel dimers, which unite after part of the C-terminal end is removed. The different dimer molecules join laterally to form anchoring fibrils. The N-terminal domains of the anchoring fibrils are bound by homology to the macromolecules in the lamina densa, stabilizing its adhesion with the underlying dermis.

Fig. 8 presents a diagram of the formation of anchoring fibrils. The pro-α1 (VII) collagen polypeptides are synthesized mainly by the keratinocytes of the epidermis, but also by the dermal fibroblasts.^52^ Three formed polypeptides are associated through their carboxy-terminal ends (NC2 domains), while their collagenous domains fold into a triple helix conformation. Two type VII collagen molecules in triple helix form antiparallel dimers and, after part of the carboxy-terminal end (NC2) is removed, the dimer formed is stabilized by intermolecular disulfide bonds.

Subsequently, several type VII collagen dimers are organized laterally in the anchoring fibrils. The binding of fibrils to the lamina densa occurs due to the non-collagenous amino-terminal domain NC1, which, by homology, binds to proteins of the lamina densa, stabilizing the association of this layer with the underlying dermis (Fig. 8).^62^, ^72^, ^75^, ^76^, ^77^

The importance of knowing the stages of the formation of anchoring fibrils lies in the fact that disturbances in any of these stages result in specific consequences for the structure and expression of the fibrils, which ultimately leads to the different phenotypes of DEB.^62^, ^72^ As will be presented below, it can be said that the DEB phenotype is, as a rule, determined by the expression and residual function of *COL7A1*, although exceptions to this pattern have already been reported.^78^, ^79^

Severe RDEB is often caused by premature stop codon variants (null variants) in both alleles of the *COL7A1*gene, resulting from nonsense substitutions, frameshift insertions or deletions, or even variants in splice sites that result in alteration in the reading phase.^7^, ^80^ Such changes cause serious consequences for the protein formed, resulting in decay of mRNA mediated by nonsense mutations (transcription degradation mechanism with premature stop codon) or in the formation of truncated polypeptides that are not able to associate for the assembly of anchoring fibrils.^7^, ^62^, ^80^

In turn, intermediate RDEB is often caused by variants in compound heterozygosis: a premature stop codon variant and an amino acid substitution.^7^ It should be noted, however, that several other types of variants, in different combinations, have also been associated with this subtype.^67^, ^79^, ^81^, ^82^, ^83^, ^84^ The intermediate form is characterized the presence of at least one variant that allows for some type VII collagen production, thus enabling the assembly, even if partial, of the anchoring fibrils. Thus, the genetic changes associated with this subtype can affect the association of polypeptides, the formation and stability of the triple helix, or cause some conformational modification in the protein.^7^, ^62^ The polypeptides formed in these cases are still capable of forming a small number of anchoring fibrils, even if unstable, which explains the more attenuated phenotype in relation to the severe subtype of the disease.^7^, ^85^

Similarly, localized RDEB is correlated to variants that minimally affect the formation of anchoring fibrils, either by reducing their expression or through slight conformational or stability changes.^86^, ^87^, ^88^ Interestingly, the most described variants in cases of localized RDEB are those that occur in splice sites.^83^, ^86^, ^88^, ^89^ Splice variants are associated with a diversity of phenotypes, depending on their influence on the sequence of the protein formed. In the localized form, they usually result in the excision of whole exons (exon skipping), without altering the remaining protein sequence. Studies have shown that these variants do not alter the totality of the formed transcripts, but only a portion of them, allowing the formation of normal transcripts to a considerable extent. This allows the assembly of the anchoring fibrils with only small functional defects, which justifies the very mild phenotype.^86^, ^88^, ^89^, ^90^

The dominant form of DEB usually involves substitutions of glycine in the collagenous triple helix domain, although other substitutions, insertions, deletions, and splice site variants have also been described.^7^, ^62^, ^91^, ^92^ These variants affect amino acids that are essential for the structure of the triple helix and, therefore, are associated with the decreased stability of the anchoring fibrils.^7^, ^62^, ^72^ Some amino acids are positioned in segments that are more important to the structure of collagen VII, and some substitutions affect the formation of the molecule more significantly than others. Thus, the nature and position of the genetic variation are closely correlated to the resulting phenotype.^7^, ^93^

## Kindler’s epidermolysis bullosa

KEB, formerly known as Kindler syndrome, was only recently classified as one of the main types of EB, based on the finding that the clinical and biological bases involved are compatible with it.^2^, ^3^, ^27^ It is a rare subtype of EB, reported in approximately 250 individuals worldwide since its first description^2^ The clinical presentation of KEB can mimic several subtypes of EB, hindering diagnosis in many cases.^27^, ^94^, ^95^ KEB was differentiated from the other three types of EB due to its peculiar characteristics. Firstly, unlike the other types, in KEB the cleavage of the skin can occur in multiple layers (intraepidermal, junctional, or sublamina densa), preventing its inclusion within one of the other groups of the disease. Moreover, distinct clinical features are very common in KEB, especially poikiloderma, which combines hyper- or hypo-pigmentation of the skin and the occurrence of clusters of blood vessels just below the skin (telangiectasias), and photosensitivity, which manifests itself as erythema and sunburn.^3^, ^27^, ^96^ The characteristic phenotype of KEB is usually progressive and includes, in addition to poikiloderma and photosensitivity, blistering and extensive skin atrophy. Extracutaneous findings in KEB include gum erosions and ocular, esophageal, gastrointestinal, and genitourinary involvement.^3^, ^97^

KEB has autosomal recessive inheritance and is caused by changes in the *FERMT1*gene, which encodes kindlin-1, a protein associated with integrins and focal adhesions.^98^, ^99^ In the skin, kindlin-1 is located next to the basal keratinocytes and acts as a protein that adapts the focal adhesions, linking the actin filaments to the membrane proteins.^97^, ^100^ In KEB patients, keratinocytes become disorganized, losing their characteristic structure and polarization, and their proliferation is reduced. *In vitro* studies have shown that the loss of kindlin-1 function is associated with abnormal cell format and changes in cell adhesion, proliferation, and motility processes.^97^, ^101^ These alterations result from disturbances in focal adhesions that, in normal situations, ensure the anchoring of the cytoskeleton to the integrin signaling platforms.^102^, ^103^ Kindlin-1 makes up the β1 integrin adhesion complex and participates in the formation of focal adhesions, which explains the consequences of the loss of its function for the structure and adhesion of skin cells and justifies the associated skin fragility phenotype.^94^, ^97^, ^99^

A total of 84 different pathogenic variants have already been described in *FERMT1*(HGMD database - www.hgmd.cf.ac.uk), most of which are associated with the formation of a premature stop codon and the consequent loss of function of kindlin-1. However, other variants have also been identified, including amino acid substitutions, splice site variants, and intronic variants.^94^, ^95^, ^97^, ^99^ Since most of the variants are null, resulting in the absence of kindlin-1, genotype-phenotype correlations are not well established for KEB, since the nature or position of the variant does not appear to significantly influence the clinical presentation. Although clinical signs of KEB are common to patients with null variants, there is an evident clinical variability, suggesting an influence of environmental and/or epigenetic factors.^97^

## Other skin fragility disorders

Skin fragility, in addition to being characteristic of the classic subtypes of EB, is also present in other groups of inherited diseases, such as squamous, erosive, hyperkeratosic, and connective tissue disorders, shown in Table 4.^2^ These diseases are similar to EB regarding the alterations in the skin layers and/or in the pathogenic mechanisms involved and, therefore, should be considered for differential diagnosis, especially in cases of newborns.^2^

**Table 4:** Other skin fragility disorders.

Skin cleavage level	Denomination	Inheritance pattern	Altered gene (protein)
Intraepidermal	Peeling skin disorders	AR	*TGM5*(Transglutaminase 5)
			*CSTA*(Cystatin A)
			*CTSB*(Cathepsin B)
			*SERPINB8* (Serpin B8)
			*FLG2*(Filaggrin 2)
			*CDSN* (Corneodesmosin)
			*CAST* (Calpastatin)
			*DSG1*(Desmoglein 1)
			*SPINK5* (LEKTI)
Intraepidermal	Erosive skin fragility disorders	AR	*DSP* (Desmoplakin)
			*JUP*(Plakoglobin)
			*PKP1*(Plakophilin 1)
			*DSC3*(Desmocollin 3)
			*DSG3*(Desmoglein 3)
Intraepidermal	Hyperkeratotic disorders with skin fragility		
	Keratinopathic ichthyosis	AD	*KRT1* (Keratin 1)
			*KRT10*(Keratin 10)
			*KRT2*(Keratin 2)
		AR	*KRT10*(Keratin 10)
	Pachyonychia congenita	AD	*KRT6A*(Keratin 6A)
			*KRT6B*(Keratin 6B)
			*KRT6C*(Keratin 6C)
			*KRT16*(Keratin 16)
			*KRT17*(Keratin 17)
Dermal	Syndromic connective tissue disorder with skin fragility	AR	*PLOD3*(Lysyl hydroxylase 3)

Modified table from Has et al. (2020) (2).

AD, autosomal dominant; AR, autosomal recessive.

Although not included as classical EB, the marked phenotypic characteristics of these disorders are skin fragility and, often, mucous membranes fragility, with the same clinical signs and health needs. Thus, for medical and socioeconomic assistance purposes, it is recommended to consider such disorders together with the classic forms of EB.^2^

## Diagnosis of inherited epidermolysis bullosa

As presented herein, EB comprises four main forms and multiple clinical subtypes, which are characterized by a wide variety of clinical signs. Periodically, researchers from around the world convene to update the diagnostic criteria and the classification of EB subtypes.^2^, ^3^, ^27^, ^104^^,^^105^ The classification aims to distinguish, in different groups, the entire genetic and clinical heterogeneity of the disease, in order to assist the precise diagnosis and subclassification. However, many of the clinical signs overlap among the subtypes, evidencing that the classification of EB, in most cases, is not so obvious.^3^, ^79^ In reality, what is noticeable within the different forms of EB is a continuous spectrum of phenotypic severity. More severe cases are, in general, easier to classify, while the cases that are distributed along this continuum require additional analysis beyond mere clinical observation.

Given the numerous protein complexes that closely interact in order to provide integrity and adhesion of the skin layers, it is to be expected that different genetic alterations affect each of these components in a different way, resulting in different consequences for the structure of the skin, ultimately resulting in the various known phenotypes of EB.^5^, ^6^, ^106^ A precise diagnosis and the correct subclassification of EB allow a prognosis of the severity of the disease, genetic counseling for the patient and family, prenatal or pre-implantation genetic diagnosis, inclusion in clinical trials, and precision medicine.^6^ EB diagnosis can be confirmed by: a) immunofluorescence mapping, based on skin biopsy analysis; b) transmission electron microscopy, through the analysis of skin ultrastructure; or c) direct genetic analysis of genes associated with EB.^1^, ^6^

Immunofluorescence mapping (IFM), or immunomapping, is one of the methods used to determine the main type of EB and, often, the probable protein that is altered.^27^ The method is based on the use of monoclonal antibodies against proteins of the dermoepidermal junction, which allows identifying the skin layer where cleavage occurs and the relative abundance of proteins.^107^ It is an important diagnostic tool, especially in cases of greater disease severity where the expression of the altered protein is absent or very reduced.[107,108] In less severe cases, skin cleavage may not be identified in the sample and, likewise, alterations in the immunoreactivity of the markers used may not be observed, leading to frequent inconclusive results.^107^

Transmission electron microscopy (TEM) allows the detection and semiquantitative evaluation of structures present in the layers of the skin, such as keratin filaments, desmosomes, hemidesmosomes, anchoring filaments, and anchoring fibrils. Thus, TEM allows the identification of changes in these structures in relation to their quantity and appearance, which are correlated to the different subtypes of EB.[27,108] Nonetheless, interpretation of the results requires significant knowledge about cell-cell and matrix-cell adhesion structures and their appearance on normal skin and on EB skin.^6^ In addition, there are few laboratories in the world that perform TEM for the analysis of EB; therefore, this technique is little used for the diagnosis of the disease.^27^

The genetic test provides the definitive diagnosis of the EB subtype, the associated inheritance pattern, and the causal pathogenic variant.^27^ Three main methods are used for genetic analysis: a) Sanger sequencing; b) sequencing of a panel of genes through next-generation sequencing (NGS); c) sequencing of the entire exome by NGS.^6^ Sanger sequencing is an excellent approach when the candidate gene is known, which can be inferred from previous tests (such as IFM and TEM), or which can be suggested when the phenotype is clearly typical of a specific subtype. When there is no definition of the candidate gene, Sanger sequencing becomes a very laborious and costly method, as it involves the analysis of all exons of the genes under analysis.^1^, ^6^ A gene panel performed by NGS allows the rapid analysis of multiple genes simultaneously, being an effective approach when the candidate gene is not known.^1^, ^6^ Complete exome sequencing allows the analysis of most protein coding regions, and is a useful tool when the candidate gene is not known. The advantage of this method over the gene panel is the possibility of discovering new genes associated with the disease. The disadvantage refers to the greater complexity for analyzing and interpreting the data, requiring additional expertise and computational resources, which increases the time until the final diagnosis.^6^

## Final considerations

EB is a heterogeneous disease that involves a wide spectrum of severity, different inheritance patterns, and many causal genes. The most severe forms of the disease have debilitating clinical signs, such as limb mutilation, recurrent inflammation and infections, chronic pain, and early lethality. Although there is still no cure for EB and treatments are only symptomatic, studies have indicated potential future therapeutic approaches.

Clinical and genetic studies have made it possible to understand many of the molecular mechanisms responsible for the adhesion of the skin layers that are associated with the different subtypes of EB. At least 16 genes are already known, and the most recent classification of the disease described a total of 34 clinical subtypes. Nonetheless, some gaps remain to be filled about the clinical and genetic foundations of EB and the mechanisms of modulation of skin fragility phenotypes. Future studies are important for a better understanding of EB and for the development of potential treatments.

## Financial support

None declared.

## Authors’ contributions

Luiza Monteavaro Mariath: Elaboration and writing of the manuscript; critical review of the literature.

Juliana Tosetto Santin: Approval of the final version of the manuscript; critical review of the literature.

Lavínia Schuler-Faccini: Approval of the final version of the manuscript; critical review of the manuscript.

Ana Elisa Kiszewski: Approval of the final version of the manuscript; critical review of the manuscript.

## Conflicts of Interest

None declared.

## CME Questions

1. EB is classified into four main types, which relate to the layer of the skin in which blisters occur. Check the alternative that correctly correlates the type of EB and the respective layer of the skin where the cleavage occurs.

a) EB**S**– intra-epidermal; JEB – lamina lucida of the basement membrane; DEB – mixed cleavage pattern; KEB – upper layer of the dermis.

b) EB**S** – upper layer of the dermis; JEB – lamina lucida of the basement membrane; DEB – intraepidermal; KEB – mixed cleavage pattern.

c) EB**S** – lamina lucida of the basement membrane; JEB – intraepidermal; DEB – upper layer of the dermis; KEB – mixed cleavage pattern.

d) EB**S**– intra-epidermal; JEB – lamina lucida of the basement membrane; DEB – upper layer of the dermis; KEB – mixed cleavage pattern.

2. Cleavage at the epidermal level of the skin, the greatest diversity of clinical subtypes, predominantly autosomal dominant inheritance pattern, the highest incidence and prevalence among types of EB. The characteristics mentioned above refer to which main type of EB?

a) EB**S**

b) JEB

c) DEB

d) KEB

3. These are cytoskeletal proteins that provide mechanical stability to epithelial cells and tissues. They associate in a heterodimer, forming the intermediate filaments that allow cell-cell binding of keratinocytes and binding of keratinocytes to the underlying layer. Which proteins does the text refer to?

a) Plectin and BP230

b) Collagen VII and XVII

c) α6β4 and α3β1 integrins

d) Keratins 5 and 14

4. Which of the following presents the proteins that act at the dermoepidermal junction and are associated with JEB?

a) Collagen VII

b) Keratin 5, keratin 14, plectin, Kelch-like 24, exophyllin-5.

c) Laminin 332, collagen XVII, α6β4 integrin, α3β1 integrin

d) Kindlin-1

5. Characterized by cleavage of the skin in the region of the sublamina densa in the upper portion of the dermis, this form of EB presents both an autosomal dominant and a recessive inheritance pattern. It is associated with a wide phenotypic and severity spectrum, ranging from the isolated occurrence of mild nail dystrophy to the generalized formation of blisters with mutilating scarring of the hands and feet, severe extracutaneous involvement, and premature death. The above description refers to which main type of EB?

a) EB**S;**

b) JEB;

c) DEB;

d) KEB.

6. It is the most severe form of DEB and its main clinical signs involve the formation of generalized blisters throughout the body, pseudosyndactyly, chronic ulcers that are difficult to heal, anemia, developmental delay, mucosal involvement, and greater susceptibility to the development of squamous cell carcinoma. The above sentence refers to which subtype of EB?

a) Recessive DEB, localized

b) Recessive DEB, severe

c) Dominant DEB, intermediate

d) JEB, severe

7. DEB is caused by alterations in a single gene, which results in morphological changes or reduction/absence of an important constituent of the skin layers, which provides integrity for the association of the epidermis with the underlying dermis. Check the alternative that indicates the protein associated with DEB and the adhesion structure formed by it.

a) Collagen XVII, anchoring fibrils

b) Collagen VII, intermediate filaments

c) Collagen VII, anchoring fibrils

d) Collagen XVII, collagen fibers

8. It is known that the type of genetic alteration and its consequence for protein expression are directly linked to the phenotypic severity of recessive dystrophic EB (RDEB). Check the incorrect alternative.

a) The presence of premature stop codon variants (null variants) in both alleles of the *COL7A1*gene is often associated with the complete absence of collagen VII expression, resulting in RDEB, severe.

b) RDEB, localized is usually associated with variants that minimally affect the formation of anchoring fibrils, either by reducing its expression or through slight conformational or stability alterations, which explains the attenuated phenotype of this subtype of EB.

c) In RDEB, intermediate, usually at least one of the alleles carries a variant that allows for some type VII collagen production, thus enabling the assembly, even if partial, of the anchoring fibrils.

d) RDEB, severe is mainly caused by glycine substitutions that are essential for the structure of the triple helix of collagen VII and, therefore, are associated with the decreased stability of the anchoring fibrils.

9. It is a rare subtype of EB, caused by changes in the *FERMT1*gene, and is associated with typical clinical signs such as poikiloderma and photosensitivity. The phrase refers to what main type of EB?

a) EB**S**

b) JEB

c) DEB

d) KEB

10. Which of the analysis methods below is not used for the diagnosis of epidermolysis bullosa?

a) Karyotype

b) Transmission electron microscopy

c) Immunofluorescence mapping

d) Next-generation sequencing

ANSWERS

**Severe bacterial skin infections. An Bras Dermatol. 2020;95(4): 407-17.**

**Table:** 

1. d	3. d	5. d	7. d	9. a
2. a	4. a	6. d	8. c	10. d
